# To Be or Not to Be: The Divergent Action and Metabolism of Sphingosine-1 Phosphate in Pancreatic Beta-Cells in Response to Cytokines and Fatty Acids

**DOI:** 10.3390/ijms23031638

**Published:** 2022-01-31

**Authors:** Ewa Gurgul-Convey

**Affiliations:** Institute of Clinical Biochemistry, Hannover Medical School, Carl-Neuberg-Str. 1, 30625 Hannover, Germany; gurgul-convey.ewa@mh-hannover.de

**Keywords:** inflammation, type 1 diabetes, type 2 diabetes, beta-cells, cytokines, free fatty acids, lipotoxicity, sphingolipids, sphingosine-1 phosphate

## Abstract

Sphingosine-1 phosphate (S1P) is a bioactive sphingolipid with multiple functions conveyed by the activation of cell surface receptors and/or intracellular mediators. A growing body of evidence indicates its important role in pancreatic insulin-secreting beta-cells that are necessary for maintenance of glucose homeostasis. The dysfunction and/or death of beta-cells lead to diabetes development. Diabetes is a serious public health burden with incidence growing rapidly in recent decades. The two major types of diabetes are the autoimmune-mediated type 1 diabetes (T1DM) and the metabolic stress-related type 2 diabetes (T2DM). Despite many differences in the development, both types of diabetes are characterized by chronic hyperglycemia and inflammation. The inflammatory component of diabetes remains under-characterized. Recent years have brought new insights into the possible mechanism involved in the increased inflammatory response, suggesting that environmental factors such as a westernized diet may participate in this process. Dietary lipids, particularly palmitate, are substrates for the biosynthesis of bioactive sphingolipids. Disturbed serum sphingolipid profiles were observed in both T1DM and T2DM patients. Many polymorphisms were identified in genes encoding enzymes of the sphingolipid pathway, including sphingosine kinase 2 (SK2), the S1P generating enzyme which is highly expressed in beta-cells. Proinflammatory cytokines and free fatty acids have been shown to modulate the expression and activity of S1P-generating and S1P-catabolizing enzymes. In this review, the similarities and differences in the action of extracellular and intracellular S1P in beta-cells exposed to cytokines or free fatty acids will be identified and the outlook for future research will be discussed.

## 1. Introduction

Diabetes mellitus is a multifaceted metabolic disease, characterized by chronic hyperglycemia and inflammation, associated with dysfunction and death of pancreatic beta-cells which supply our body with a glucose-lowering hormone insulin [1,2,3,4]. Though meanwhile a number of diabetes subtypes have been recognized, the main two forms are the autoimmune-mediated type 1 diabetes (T1DM) and the metabolic syndrome-induced type 2 diabetes (T2DM).

In the recent decades disturbances in sphingolipid metabolism have been linked to diabetes development and beta-cell failure [5,6,7,8,9,10,11,12,13,14]. Sphingolipids (SLs) are crucial components of cellular membranes and are involved in the cell survival, proliferation, differentiation and apoptosis [6,15,16]. Sphingosine-1-phosphate (S1P) is the only SL that lacks the structural function and acts exclusively as a bioactive mediator [6,15], and will stand in the center of this review. S1P exerts multiple biological effects both intracellularly as a second messenger and/or epigenetic regulator as well as extracellularly by binding to the specific G-protein-coupled receptors [15,17,18,19,20,21,22]. S1P metabolizing enzymes are highly regulated by oxidative stress and inflammatory conditions [15,23]. In pancreatic beta-cells, S1P has been shown to regulate glucose-stimulated insulin secretion and sensitivity of pancreatic beta-cells to T1DM- and T2DM-simulating conditions [5,11,12,24,25,26,27,28,29,30,31].

In this review various aspects of S1P metabolism and action in pancreatic beta-cells will be addressed. The effects of T1DM- vs. T2DM-simulating conditions on S1P metabolizing enzymes and S1P biosynthesis in beta-cells will be presented. Finally, the divergent role of S1P in beta-cells under T1DM and T2DM conditions will be discussed.

## 2. Overview of Mechanisms of Beta-Cell Destruction in T1DM

Type 1 diabetes mellitus (T1DM) is a serious autoimmune disease with a strong genetic background characterized by a progressive loss of pancreatic beta-cells, resulting in absolute insulin insufficiency requiring life-long substitution [3,32,33]. The autoimmune process is initiated by yet not fully characterized triggers (virus infections, dietary factors, others) and executed by activated immune cells [3,32,34]. The infiltrating immune cells secrete reactive oxygen species (ROS), nitric oxide (NO), proinflammatory cytokines and chemokines resulting in islet inflammation (insulitis) [35].

The major proinflammatory cytokines involved in beta-cell failure during T1DM development are IL-1β, TNFα and IFNγ [1,4,32,36], which stimulate pleiotropic effects by receptor-mediated mechanisms [37,38,39,40,41,42]. Multiple molecular mechanisms have been associated with beta-cell death in T1DM including mitochondrial and endoplasmic reticulum (ER) stress responses, ROS generation and induction of NO production (only in rodent beta-cells), impaired calcium homeostasis and disturbed autophagy [30,32,40,41,43,44,45,46,47,48,49,50,51,52,53,54,55,56]. IL-1β and TNFα stimulate ROS production particularly in mitochondria, which are in beta-cells characterized by a very low detoxification capacity of hydrogen peroxide toxicity [46,52,53,57]. Overexpression of hydrogen-peroxide detoxifying enzyme catalase has been shown to protect beta-cells against cytokine toxicity [46,52] with no detrimental effects on glucose-stimulated insulin secretion (GSIS) [58]. Proinflammatory cytokines also affect the expression of genes involved in inflammatory pathways and induce proinflammatory signaling [2,4,35,38,43,56,59,60,61,62,63,64,65,66,67,68,69,70]. Finally, cytokines dysregulate insulin biosynthesis and glucose-stimulated insulin secretion (GSIS) [30,49,54,55,56,71,72]. 

Interestingly, a number of new investigations suggest that dietary fats and disrupted sphingolipid tissue profiles may be considered as triggers that could induce or accelerate the autoimmunity onset in T1DM [73]. Polymorphisms in several genes encoding enzymatic machinery of the sphingolipid metabolism were linked to overt T1DM [5]. Moreover, profound changes in sphingolipid serum profiles upon autoimmunity development were detected in T1DM individuals [5,11,12,13,14,74,75,76]. While the role of these changes in plasma sphingolipids on beta-cell fate remains unclear, it has been shown that fingolimod (FTY-720), a functional antagonist of S1P1, can prevent islet infiltration and diabetes development in the animal models of autoimmune diabetes [77,78,79,80,81]. This protective effect was associated with a reduced expression of proinflammatory markers in beta-cells, indicating that targeting the S1P action in beta-cells might be used as a protective strategy.

## 3. Overview of Mechanisms of Beta-Cell Destruction in T2DM

The majority of patients suffer from type 2 diabetes (T2DM) that is triggered by an unhealthy lifestyle together with a genetic predisposition [1,2,82,83,84,85]. The diagnosis of T2DM is preceded by prediabetes, which is characterized by glucose intolerance and low-grade systemic inflammation [1,83]. During a relatively long phase of prediabetes, beta-cell proliferation is increased, leading to elevation of beta-cell mass in attempt to fulfill body’s needs for insulin [1]. Beta-cells face a multifaceted cellular stress response due to accelerated insulin biosynthesis and secretion in attempt to fulfill insulin demand. This stress response is potentiated by high levels of circulating free fatty acids (FFAs), hyperglyceamia (HG) and inflammatory mediators [1,2,4,82,85,86].

Under physiological conditions FFAs potentiate GSIS [2,4]. However, chronic exposure to FFAs has been associated with disturbed GSIS and beta-cell apoptosis [4,85,86]. Several molecular mechanisms are believed to contribute to lipotoxicity in beta-cells, such as changes of the expression of multiple proteins involved in glucose uptake, metabolism and insulin secretory capacity, generation of ROS, ER and mitochondrial stress, calcium disturbances or biosynthesis of complex lipid species [1,4,86]. 

Various FFAs affect beta-cell function and fate differentially, with a predominance of toxic effects of saturated FFAs (such as palmitate, PA) and very long-FFAs [85,86,87]. Monounsaturated FFAs, such as oleate (OA), have been shown to counteract the toxicity of saturated FFAs in rodent beta-cells [4], however recent findings indicate that OA might be the most toxic physiological FFA in human EndoC-βH1 beta-cells [87,88,89,90]. Chronic exposure to high concentrations of FFAs has been associated with a decreased gene expression of insulin and downregulation of the insulin secretory capacity [91]. Human beta-cells are characterized by a distinct sensitivity to FFAs comparing with rodent beta-cells [89,90,92]. This broader susceptibility of human beta-cells to a variety of FFAs, including OA, is not fully understood. Recently stearoyl-CoA desaturase 1 (SCD1), an ER enzyme that synthesizes monounsaturated fatty acids from PA and stearic acid (SA), was shown to be abundantly expressed in human beta-cells [87]. PA downregulates SCD1 expression [87]. The siRNA-mediated SCD1 silencing or chemical inhibition of its activity were shown to impair autophagy and induce beta-cell death [87,93]. These findings indicate that the biological availability of intracellular PA for various metabolic pathways may be essential for sensitivity of human beta-cells to lipotoxicity.

PA is the main substrate for sphingolipid biosynthesis [6,8]. Many lines of research point to an important role of sphingolipids in the development of beta-cell dysfunction and T2DM [6,7,10,25,94,95,96,97,98]. Particularly proapoptotic ceramides and S1P seem to affect many aspects of beta-cell fate during T2DM development (reviewed in [10,94]). Interestingly, a distinct sphingolipid serum and tissue profile of T2DM patients as compared to healthy individuals have been reported [97,99,100,101,102,103,104]. The elevated plasma concentration of S1P coincidence with insulin resistance, obesity and hyperinsulinemia [100,104].

## 4. Sphingosine-1 Phosphate Metabolism, Receptors and Transporters in Pancreatic Beta-Cells

**Figure 1:**
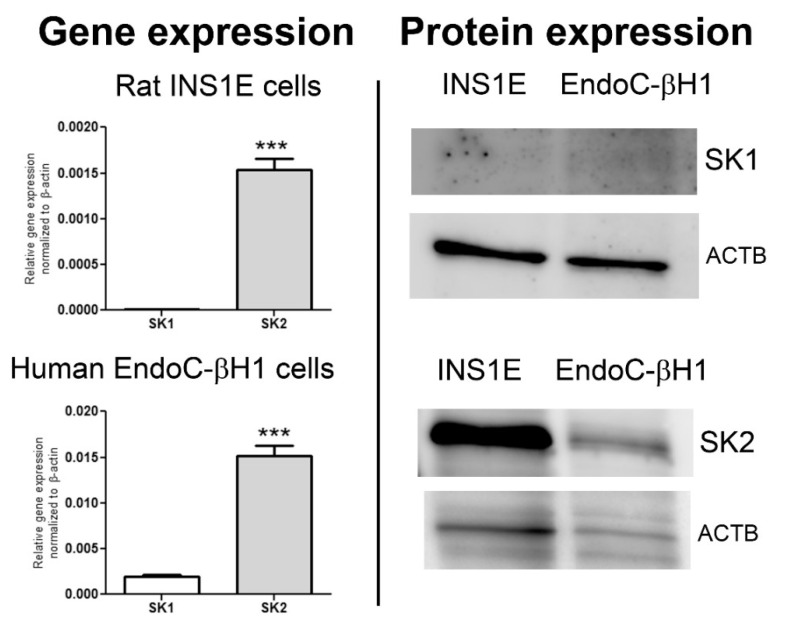
Gene and protein expression of sphingosine kinases 1 and 2 in rat INS1E and human EndoC-βH1 beta-cells. Total RNA was extracted from untreated rat INS1E (a kind gift of Prof.C Wollheim, Geneva, Switzerland) and human EndoC-βH1 beta-cells (ENDOCELLS SARL, Paris, France) (RNeasy Kit, Qiagen, Hilden, Germany). Cells were cultured in a humidified atmosphere at 37 °C and 5% CO_2_ as described [31], and were free from mycoplasma contamination.The quality of RNA was verified by agarose gel electrophoresis. RNA was quantified spectrophotometrically at 260/280 nm. Thereafter, 2 µg of RNA were reverse transcribed into cDNA using a random hexamer primer (Thermo Fisher Scientific, Braunschweig, Germany) and RevertAid H Minus M-MuLV reverse transcriptase (Thermo Fisher Scientific). QuantiTect SYBR Green^TM^ technology (Qiagen) was employed. The reactions were performed using rat (rSK1 fw-CTTCTGGAGGAGGCTGAGGT, rev- TCAGACCGTCACCGGACAT; rSK2 fw-CAAGCCCTACACATACAGCG, rev-GCCACGTGGGTAGGTGTAGA, rActin-b fw-GAACACGGCATTGTAACCAACTGG, rev-GGCCACACGCAGCTCATTGTA) and human (huSK1 fw-TGGGACGCTCTGGTGGTCATGT, rev-TACACAGGGGCTTCTGGATGGC, huSK2 fw-TGCTCCATGAGGTGCTGAACGG, rev-AATCCCCCGTGCTGGTTCACTG, huActin-b fw-ATGGATGATGATATCGCCGC, rev-TTCTGACCCATGCCCACCA) specific primers (Microsynth, Balgach, Switzerland) on a ViiA7 real-time PCR system (Thermo Fisher Scientific) with the following protocol: 50 °C for 2 min, 95 °C for 10 min, and 40 cycles comprising a melting step at 95 °C for 15 s, an annealing step at 62 °C for 60 s and extension step at 72 °C for 30 s. The quality of reactions was controlled by analysis of melting curves. Each sample was amplified in triplicate. Data normalization was performed against the housekeeping gene β-actin. Statistical analysis was performed using *t*-test, *** *p* < 0.001.Total cell protein was collected in ice-cold PBS containing a cocktail of protease inhibitor (Roche, Mannheim, Germany) and followed by sonication. Protein concentration was determined by BCA assay (Thermo Fisher Scientific). Following denaturation, 50 µg of samples of rat INS1E or human EndoC-βH2 beta-cells were separated onto 12.5% gels, blotted onto nitrocellulose and blocked with 5% dry-fat milk as described [31]. Primary antibodies against SK1 sc-48825 (M209) (Santa Cruz, Heidelberg, Germany), SK2 17096-1-AP (Proteintech, Manchester, UK), beta-actin (ACTB) sc-47778 (C4) (Santa Cruz) were used at the dilution 1:500, secondary peroxidase conjugated Affini Pure IgG (H + L) at the dilution 1:2000. The hybrids were visualized using the enhanced chemiluminescence detection kit and captured by the INTAS chemiluminescence detection system (Intas Science Imaging Instruments, Göttingen, Germany).

S1P is biosynthesized from sphingosine, an amino alcohol with an 18-carbon unsaturated alkyl chain, which acts as a backbone for all sphingolipids [6,15]. Sphingosine can be quickly released from membrane sphingomyelin by the action of sphingomyelinases and/or ceramidases, or can be produced de novo by a series of reactions initiated by serine-palmitoyl-transferase (SPT) in the ER from palmitoyl-CoA and L-serine [6,15]. The reaction of sphingosine phosphorylation is catalyzed by sphingosine kinases. Sphingosine kinase 1 (SK1) and sphingosine kinase 2 (SK2) are characterized by similar amino acid sequences, but differ substantially in their intracellular localization, regulation, and function [6,15]. The SK1-catalyzed phosphorylation of sphingosine at the plasma membrane is the central site of S1P production [100], however in the pancreatic beta-cell, SK1 is rather weakly expressed [10,30,105,106]. SK2 induces S1P generation in the ER, mitochondria and nucleus [6,15]. As shown in Figure 1 by real-time PCR as well as Western blot measurements, in pancreatic beta-cells of rat and human origin, SK2 is the predominant isoenzyme.

S1P can be recycled by the action of ER-localized S1P phosphatases (SPP1 and SPP2) into sphingosine [6,15,107]. Though both isoforms are expressed in rodent beta-cells, the predominant form is SPP2 [30]. Thus far, no data on the expression of SPP1 and SPP2 are available from human beta-cells.

Additionally, dephosphorylation of S1P can be also catalyzed by several lipid phosphate phosphohydrolases (LPPs), which are membrane-associated enzymes with their active sites located on the outer surface of the plasma membrane or at the lumenal surface of Golgi and endosomes [108,109,110,111,112]. The expression and action of LPPs in beta-cells has not been fully characterized. Though rodent beta-cells express receptors for lysophospholipids the RNAseq data show a low–moderate level of LPP expression [41]. Further studies are needed especially with respect to human beta-cells.

The irreversible degradation of S1P is the last step in the sphingolipid pathway and is catalyzed by S1P lyase (SPL) [111,113]. SPL is a highly conserved pyridoxal 5′-phosphate-dependent enzyme that is localized on the outer ER leaflet [111]. SPL cleaves S1P to hexadecenal and phosphoethanolamine. The products of SPL reaction can be used as glycerol and phospholipid substrates in the glycerophospholipid pathway [6,111,114], raising palmitoyl-CoA or phosphatidylethanolamine pools, respectively [109]. In rodent pancreatic beta-cells the expression of SPL is in a low/moderate range as compared to other tissues [30], while human beta-cells are characterized by a significantly higher SPL expression [31].

It has been demonstrated that S1P can act as a ligand of cell-surface receptors or as a direct intracellular modulator in various cell types [115]. The local concentration of extracellular S1P varies and depends on the specific tissue capacity of S1P biosynthesis and transport as well as on the availability of circulating S1P pool generated, e.g., by blood platelets [107]. The transport of S1P has been shown to be facilitated by the ABC transporter family and the Spns2 transporter [15,16,114]. In rodent beta-cells the predominant transporter is the Abca1, followed by Abcc1 and Spns2 [30]. Thus far, the expression pattern of S1P transporters has not been characterized in human beta-cells. The S1P receptors (S1PR1–S1PR5) belong to G protein-coupled receptors and are characterized by distinct mechanisms of action depending on the G subunit involved (excellently reviewed by [114,115]). In rat islets four types of S1P receptors (S1P1, S1P2, S1P3, S1P4) were detected; in mouse islets was also S1P5 present [105,116]. Similar results were obtained in a rat INS1 insulin-producing cell line [105]. The mRNA expression of S1PR2, 3 and 5, of which S1PR3 was the predominant subtype, was also detected in a more differentiated INS1E beta-cell line [30]. Thus far, data from human beta-cells are missing. The presence of S1PR5 which was also detected in the nervous system and spleen [115,117] could further explain many similarities in the role of S1P in neurons and pancreatic beta-cells [30,118,119,120,121,122,123]. S1PR2 and S1PR3 are coupled predominantly to Gq and activate phospholipase C (PLC) to induce Ca^2+^ mobilization through the production of inositol 1,4,5-trisphosphate [124,125], and induce activation of MAPK kinases [126]. S1PR5 was shown to interact with Gα subunits [108], that inhibit PLC activity. As intracellular targets of S1P, the CIAP2 (cellular inhibitor of apoptosis 2), CerS2, prohibitin 2 and TRAF2 (TNF receptor associated factor 2) proteins as well as human telomerase reverse transcriptase (hTERT) have been proposed [6,15,16,127,128,129]; no validation of these targets has been undertaken in beta-cells so far. The cell function can be affected by intracellular S1P also at the level of epigenetic regulation [130,131]. Nuclear S1P has the ability to directly bind and inhibit the histone deacetylases HDAC1 and HDAC2 [130,131]. Though this aspect has not been directly addressed in beta-cells so far, we observed that the mRNA expression of a number of genes is significantly influenced by SPL overexpression in INS1E cells (e.g., prohibitin 2, mimitin, Sec61α) [30]. Finally, a growing number of experimental data point to a possible role of calcium as a second messenger of intracellular actions of S1P [30,118,121]. Our own findings confirmed changes in the intracellular calcium pool upon SPL overexpression in INS1E cells [30].

## 5. Effects of Proinflammatory Cytokines on S1P Metabolism in Pancreatic Beta-Cells

The expression of S1P metabolizing enzymes as well as S1P receptors and transporters is significantly modulated by the action of proinflammatory cytokines in rodent beta-cell lines and islets [30]. Importantly, the short-term vs. prolonged incubations with cytokines elicit divergent effects, particularly in the case of S1P receptors and transporters [30].

A short exposure to proinflammatory cytokines of rat INS1E beta-cells strongly decreases the S1PR3 mRNA expression, but mildly elevates the mRNA expression of S1PR2 [30]. A prolonged exposure to cytokines results in an increased mRNA expression of all S1P receptors [30]. A similar regulation was observed in the case of S1P transporter mRNA expression [30]. However, the characterization of protein expression of S1P receptors and transporters and their distribution on the plasma membrane of beta-cells exposed to various cytokines is still missing. Similarly, the pattern of S1P receptors and transporters of human beta-cells and other islet cell types in pancreata from T1DM donors still needs to be elucidated.

Proinflammatory cytokines, particularly IL-1β, were shown to stimulate the mRNA expression of SK1 in INS1 cells and rat islets within 1–8 h after addition to cell culture medium, with a time-dependent decreasing effect [106]. A 24 h incubation with proinflammatory cytokines failed to affect the mRNA expression of SK1 [30]. In contrast, no effects on SK2 mRNA were observed in response to 1–8 h incubation with proinflammatory cytokines [106], while upregulation of the mRNA expression after 24 h exposure was detected in INS1E cells [30]. Cytokines have been also shown to enhance the activity of SK2 in rat beta-cells [105,106]. In parallel, proinflammatory cytokines diminish the mRNA expression of SPL in INS1E cells and rat islets but enhance the mRNA expression of SPP2 [30]. These observations suggest an increased rate of S1P turnover by SPP2 leading to an increased sphingosine and/or ceramide generation in beta-cells upon cytokine exposure. However, a possible upregulation of the S1P generation rate locally in mitochondria, nucleus und other specific locations cannot be excluded due to a particularly strong expression of SK2. Thus, cytokine action could foster differential subcellular S1P concentrations in beta-cells. The effects of proinflammatory cytokines on the amount S1P and other sphingolipids in beta-cells have not yet been fully characterized. It has been shown that IL-1β increases the generation of S1P in INS1 cells [105,106]. Whether these alterations might occur in human beta-cells under cytokine assault and whether they could participate in the cytokine toxicity needs further investigation.

## 6. Effects of Fatty Acids and Hyperglycemia on S1P Metabolism in Pancreatic Beta-Cells

Palmitate has been shown to mildly increase SK1 expression in INS1 and INS1E beta-cells [25]. The effects of PA on SK2 expression in beta-cells are unclear; while Veret et al. failed to observe a significant effect of PA on the SK2 mRNA and protein expression [25], in their recent paper Song et al. demonstrated a two-fold induction of SK2 mRNA and protein expression [132]. A significant upregulation of SK2 activity has been observed in MIN6 cells exposed to hyperglycemic conditions and occurred in parallel with induction of insulin secretion [27]. PA has been shown to increase intracellular S1P and its release in mouse MIN6 cells [27]. Whether SPL and/or SPP expression might be affected by palmitate in rodent and human beta-cells remains unclear. The effects of OA or other major FFAs on S1P metabolism in beta-cells, especially those of human origin, has not yet been described. Increased plasma and tissue concentrations of ceramides and S1P were observed in animal models [133] and in human T2DM individuals [99,100,103,104,134].

## 7. Effects of S1P on Cytokine Toxicity in Pancreatic Beta-Cells

Both intracellular and extracellular pools of S1P have been shown to be significantly affected in various cell types by T1DM-simulating conditions and in individuals suffering from T1DM [5,11,14,74,75]. Interestingly, data gained so far indicate that the effects of S1P on cytokine toxicity differ substantially depending on whether beta-cells are exposed to exogenous S1P or whether the beta-cell experiences fluctuations of the intracellular S1P levels.

### 7.1. Extracellular S1P

In plasma S1P produced mainly by erythrocytes and platelets (≤1 µM) exists a complex with apolipoprotein M or albumin [107]. Distinct lipidomic profiles have been associated with the age and islet autoimmunity in children who later in life progress to T1DM [5,11,14,74,75]; however so far no data are available on the local concentrations of S1P in the pancreas of T1DM individuals. The exact changes of plasma and tissue S1P have not been so far documented during the development of autoimmunity in animal models of T1DM. Nevertheless, incubation of isolated rodent islets and beta-cell lines with S1P (≤5 µM) has been associated with protection against cytokine-mediated cell death and dysfunction [30,105,106]. The cytokine-mediated TUNEL staining, cytochrome c release and caspase-3 activation were reduced in rodent beta-cells after treatment with S1P at nanomolar concentrations [105]. Similar observations have been done in murine MIN6 beta-cells exposed to TNFα in the presence of S1P [26]. Beneficial effects of S1P against cytokine toxicity were not associated with decreased cytokine-mediated iNOS expression or NO generation [105]. This observation indicates a possible important role of S1P in protection of human beta-cells against cytokine toxicity, since in human beta-cells cytokines exert their toxic effects without induction of the iNOS pathway [50,135,136]. Exposure of INS1E cells to S1P results in an increased cAMP generation [30], extending the earlier observations that S1PR2 activation induces cAMP production in other cell types [137,138,139,140]. It has been demonstrated that HDL, which is enriched in S1P through its binding to apoM, could counteract beta-cell apoptosis induced by cytokines [141]. The effects of exogenous S1P have been shown to be mediated mainly by the S1P2 or S1P3 receptors, and by the activation of the PKC pathway [26,105,142]. Importantly, exposure of INS1E cells to high concentrations of S1P (>5 µM) was shown to impair cell viability and to induce caspase-3 activation [30]. Thus, extracellular S1P, at low concentrations, seems to play a protective role against cytokine-mediated beta-cell death and dysfunction in the experiments in vitro. However, it remains unclear whether S1P could also protect beta-cells and islets in vivo under T1DM-conditions.

### 7.2. Intracellular S1P

The intracellular concentration of S1P is kept low (~nM) due to a high turnover regulated by S1P metabolizing enzymes. The basal concentration and the effects of proinflammatory cytokines on beta-cell S1P in human beta-cells under acute and chronic exposure to cytokines have not yet been described. Thus far, the role of intracellular S1P in cytokine toxicity was studied mainly by genetic modifications of SPL in rodent beta-cell lines and islets [30], and the studies in beta-cell lines with a genetically modified SK1/SK2 or SPP expression are still missing. Interestingly, the action of intracellularly produced S1P in beta-cells exposed to proinflammatory cytokines seems to be opposite to that of extracellular S1P [30].

First, the observation that cytokines (15 min–8 h incubation, IL-1β and TNFα) increase SK activity and S1P concentration in rat INS1 cells and isolated islets, suggests that a rise of S1P may participate in cytokine toxicity [106]. These data have been strengthened by a discovery of several polymorphisms in the human Sk2 gene in T1DM individuals [5].

Our data strongly indicate that intracellularly generated S1P participates in acute cytokine toxicity to beta-cells, at least in the early phase of cytokine assault (24 h) [30]. We observed that the expression level of SPL in rodent beta-cells and islets is downregulated in response to 24 h incubation with cytokines [30]. Overexpression of SPL protected insulin-secreting INS1E cells against caspase-3 activation after a 24 h exposure to proinflammatory cytokines (IL-1β, TNFα and IFNγ) [30]. This protective effect was strongly associated with the maintenance of calcium homeostasis [30]. Interestingly, prevention of cytokine-mediated apoptosis by SPL overexpression was not facilitated by reduction in the cytokine-mediated NFκB-iNOS-NO pathway [30], the classical mechanism of cytokine toxicity in rodent beta-cells [2,46,143]. Similarly, SPL overexpression failed to downregulate cytokine-induced ROS generation [30]. Instead, SPL overexpression counteracted cytokine-mediated inhibition of cell proliferation and ATP content [30]. This went in parallel with an elevated expression of ER (BiP, Sec61a) and mitochondrial (Phb2, mimitin) chaperones [30]. Moreover, SPL overexpression provided protection against cytokine-mediated CHOP upregulation and ER stress activation.

The observed changes of expression of various ER and mitochondrial chaperones in SPL overexpressing INS1E cells may indicate that changes in intracellular S1P concentrations could epigenetically regulate gene expression in beta-cells, like in other cell types [130,131]. Interestingly, though SPL overexpression has been reported to be implicated in toxic effects of hexadecenal accumulation in various cell types [144,145], in cytokine-treated INS1E beta-cells SPL overexpression provided protection, an effect most likely related to a high expression level of the enzyme responsible for hexadecenal detoxification, namely ALDH3A2 [30]. It will be important to evaluate the expression and activity of SPL in beta-cells chronically exposed to cytokines, and to investigate the effects of double-transfection approaches including SK1/SK2 and SPL or SPP in future. Additionally, the role of various S1P transporters in S1P effects should be studied to determine the role of intracellularly generated S1P in detail. In vivo studies performed in S1PR2 KO mice, which were treated with STZ to induce autoimmune diabetes, which revealed that this KO mouse model was protected from STZ-diabetes, an effect that correlated with reduced apoptosis of beta-cells and lower blood glucose [142]. These findings are in line with diabetes prevention achieved by FTY0720 in two models of autoimmune diabetes, namely the NOD mouse and the IDDM rat [77,78,80].

## 8. Effects of S1P on FFA Toxicity in Pancreatic Beta-Cells

Elevated levels of circulating S1P have been observed in obese and T2DM individuals [99,100,103,104,134], suggesting an important role of S1P in the development of these disorders. Exogenous S1P has been shown to upregulate the basal insulin secretion and potentiate GSIS [24,27,30], indicating a possible role of increased concentration of circulating S1P in the onset of hyperinsulinemia. Intracellularly generated S1P seems to modulate the beta-cell response to glucolipotoxicity with opposing effects related to the intracellular site of its biosynthesis.

### 8.1. Extracellular S1P

HG was shown to increase S1P release by stimulation of SK2 in MIN6 cells [27]. S1P secreted by beta-cells was also linked to acceleration of GSIS [24,27,106] similarly to the potentiating effect of addition of S1P to cell culture medium [30]. Incubation of beta-cells with S1P has been also shown to increase their proliferation rate, probably by stimulation of cAMP generation [30], a phenomenon which participates in the increased beta-cell mass in the metabolic state or early stages of T2DM. S1P supplementation in PA-treated INS-1 or MIN6 cells was shown to prevent cell death [28], when used at low concentrations (<1 µM). Addition of S1P to cell culture medium was shown to prevent lipotoxicity in SK1 KO mouse MIN6 beta-cells and islets isolated from SK1 KO mice [28].

On the other hand, the study with the use of JTE-013, an S1PR2 antagonist, demonstrated a partial prevention of PA-mediated apoptosis and inhibition of proliferation by this approach [25,26]. This is indicative for a possible contribution of the secreted pool of S1P generated by beta-cells in PA toxicity, as well as a possible toxic action of extracellular S1P at local high concentrations.

The duration and intra-islet concentration of S1P surrounding beta-cells might decide the net effect of S1P on beta-cell fate under exposure to FFAs/HG. Similarly, the effects of other FFAs, such as OA, the main physiological mono-unsaturated FA, have not been yet studied with regard to S1P release from beta-cells. Additionally, the data regarding the input of S1P on the toxic effects of other FFAs used alone or in combination with PA are still missing. This would be particularly of great interest with regard to human beta-cells, which are characterized by a distinct sensitivity profile to various FFAs than so-far relatively well characterized rat and mouse beta-cells (see above Section 3). Finally, the S1P secretory capacity of other islet cell types, particularly of alpha-cells, as well as of endothelial and nerve cells could also influence the fate of beta-cells under T2DM conditions.

### 8.2. Intracellular S1P

Exposure to PA + HG was shown to induce SK1 expression and activity in INS1 beta-cells, followed by a raise of dihydro-S1P concentration [25]. On the other hand, in mouse MIN5 beta-cells an upregulation of S1P release was correlated with enhanced SK2 expression [132].

A pharmacological inhibition of SK activity was shown to potentiate beta-cell apoptosis induced by PA [25]. However, the intracellular source of S1P generation seems to be crucial for the observed effects. The accumulating body of evidence indicates a prosurvival role of SK1-derived S1P in beta-cells exposed to PA and HG, whereas a proapoptotic role of S1P generated by SK2 was reported [25,28,132]. Since the expression of SK2 is much higher than that of SK1, the predominant source of intracellular S1P in beta-cells is SK2. Under increased PA availability de novo biosynthesis of sphingolipids is stimulated, and the majority of S1P is expected to be produced in beta-cell mitochondria, ER and nucleus. Therefore, not surprisingly, overexpression of SK2 was shown to accelerate PA-mediated toxicity in beta-cells [132]. Interestingly, PA stimulates nuclear export of SK2 to the cytoplasm, where SK2 mediates mitochondria-dependent apoptosis signaling via its BH3 domain [132]. Thus far, no in vitro data are available on the effects of the SK2 specific inhibitors (such as HWG-35D) on lipotoxicity induction in beta-cells.

In contrast, SK1 overexpression has been shown to protect beta-cells from PA toxicity, by a mechanism independent of the S1P receptor activation [25]. The protective effect of SK1 overexpression was associated with decreased levels of proapoptotic ceramides in INS1 beta-cells exposed to PA. Overexpression of SK1 was shown to reduce mainly C18, C24 and C26 ceramides under HG condition [25]. Additionally, SK1 overexpression resulted in prevention of PA-induced mitochondrial stress (membrane potential loss and cytochrome c release) as well as ER stress (impairment of protein trafficking between ER and Golgi). In line with these observations, suppression of SK1 enzymatic activity by dominant negative form of SK1 accelerated PA-mediated cell death in MIN6 and INS-1 beta-cells [28].

In our recent study, we investigated the role of SPL in lipotoxicity in rat INS1E and human EndoC-βH1 beta-cells [31]. As the endogenous expression of SPL is rather low in rat INS1E cells [30], we used in our studies cells overexpressing SPL. SPL overexpression potentiated PA-mediated viability loss, proliferation inhibition and ROS generation [31]. These deleterious effects went along with accelerated ER stress and imbalance in mitochondrial chaperone expression [31]. Interestingly, cells overexpressing SPL were insensitive to the protective effects of OA and this correlated with a reduced expression of *Plin2* and decreased number of lipid droplets [31]. Whether the potentiation of FFA-induced ROS formation and cellular stress might involve an increased biosynthesis of proapoptotic ceramides needs further investigation. Apparently, human beta-cells are characterized by a distinct sensitivity to various FFAs as compared to rodent beta-cells [89,90,92], and it is meanwhile well established that OA is toxic in human beta-cells [89]. Interestingly, we observed a significantly higher SPL expression in human vs. rodent beta-cells. Suppression of SPL in human EndoC-βH1 beta-cells lead to protection against FFA-mediated caspase-3 activation. These new data indicate that the degradation of intracellular S1P by SPL might be crucially involved in the regulation of toxic effects of FFAs.

The in vivo data widely confirm in vitro observations about the role of both SK isoforms. The SK1 KO mice kept on a high-fat diet develop diabetes in contrast to wild-type mice on the same diet, which develop only glucose intolerance [28,146]. This phenomenon was correlated with a dramatic reduction in beta-cell mass in HFD fed SK1 KO mice [28], which overcame the beneficial effect of SK knockdown on systemic insulin sensitivity and glucose tolerance [28]. Moreover, in vivo overexpression of SK1 gene in KK/Ay type 2 diabetic mice [147] or administration of S1P analogue to HFD-fed mice [148] were shown to provide protection against insulin resistance and T2DM development. SK2 KO mice kept on HFD significantly improve their diabetic phenotypes [132]. Finally, SPP2 KO mice were shown to exhibit glucose intolerance due to the defective adaptation of pancreatic beta-cell mass [29].

Overall, both the subcellular localization of S1P biosynthesis as well as the cellular degradation capacity of S1P seem to play a crucial role in lipotoxicity in beta-cells. It will be important to determine the role of various S1P receptors and S1P transporters in beta-cells under lipotoxic stress. Additionally, inhibitors of SPL need to be investigated for their potential therapeutic effects in beta-cells under T2DM development. Certainly beta-cell specific mouse models of SK1, SK2, SPP and SPL treated with HDF would allow assessment of a specific role of these enzymes and S1P for diabetes development.

Finally, a potential role of S1P in human islets and -more importantly human beta-cells in the context of obesity or T2DM is still missing. The observations of PA and PA + HG on S1P metabolism and action should be broadened by experiments involving other FFAs, particularly the major toxic FFA for human beta-cells, OA [89].

## 9. Similarities and Differences in S1P Action in Beta-Cells under T1DM and T2DM

Though no detailed data exist so far regarding the expression pattern of S1P receptors, transporters and metabolic enzymes in human pancreas, the in vitro and in vivo studies indicate that S1P may be implicated in the regulation of insulin biosynthesis, secretion and beta-cell fate under conditions simulating T1DM and T2DM development.

Thus far, it has been shown in rodent (mouse and rat) beta-cells and islets that extracellular S1P in nanomolar concentrations displays protective effects against both cytokine and PA-mediated toxicity (Table 1), though the effects of chronic exposure (>48 h) have not been yet characterized.

Intracellular S1P appears to have distinct effects in beta-cells treated with cytokines vs. PA/HG (summarized in Table 2). This strongly depends on cytokine vs. PA/HG mediated changes in the expression of S1P metabolizing enzymes, but also on the distinct molecular mechanisms involved in cytokine vs. PA toxicity.

In rat beta-cells, SK1 and SK2 were shown to be involved in either insulin biosynthesis and/or GSIS [24]. No effects of SPL overexpression on GSIS in the presence or absence of cytokines were observed in INS1E cells [30]. The role of S1P-modulating enzymes in cytokine- or FFA-mediated effects on insulin biosynthesis and secretion in human beta-cells remains to be elucidated.

Studies performed in the SK1 KO mouse model revealed a predominant protective effect of SK1 in prevention of beta-cell mass loss under high-fed diet (HFD) conditions [25,28]. SK2 KO mice are protected against HFD-induced diabetes development [132]. The characterization of susceptibility of the SK1 and SK2 KO mouse models to STZ-induced or virus-induced autoimmune diabetes is still missing. Interestingly, fingolimod (FTY-720) was shown to reverse autoimmune diabetes development in IDDM rat and NOD models [77,78,80] as well as HFD-induced insulin resistance and weight gain in C57VL/6 mice [148]. Therefore, it seems that the overall effects of exogenous S1P strongly depend on its local concentration, tissue distribution and duration of exposure.

Cytokines and PA were shown to induce SK activity in INS1 cells and rat islets [27,106], however with major differences in time-response and its consequences. While cytokine-mediated S1P formation was not followed by its release at least in rodent beta-cells [106], PA and HG were shown to induce S1P secretion [27]. As exogenous S1P was shown to potentiate GSIS, such a PA-mediated or HG-dependent release of S1P could possibly contribute to insulin hypersecretion in the metabolic state preceding T2DM, a phenomenon not observed during T1DM.

The unique expression profile of SK enzymes in beta-cells might have important consequences for beta-cell survival and function, since the distinct localization sites of SKs are linked to differential function of each isoform. The beta-cell is well equipped with the proapoptotic SK2, while the expression of SK1 is very low [25,30], the phenomenon which we confirmed in human EndoC-βH1 beta-cells in the present study. The in vitro and in vivo studies on the role of SK1 and SK2 in cytokine-mediated beta-cell dysfunction and death are still missing in contrast to multiple studies on the role of both isoforms in context of gluco/lipotoxicity [25,27,28,132]. It is meanwhile well established that SK1-derived S1P protects beta-cells against PA and HG/PA toxicity, while SK2-derived S1P accelerates glucolipotoxicity. It seems plausible that the high expression of SK2 might also play a crucial role in cytokine toxicity, though this hypothesis requires experimental verification.

The role of S1P degrading enzyme SPL has been less studied. Our group showed that the relatively low expression of SPL contributes to cytokine toxicity in the early phase of cytokine assault (24 h) [30]. In INS1E beta-cells SPL overexpression counteracts cytokine-induced ER and mitochondrial stress responses, without affecting the NFkB-iNOS and ROS pathways [30]. Recently we have shown that human beta-cells are characterized by a significantly higher expression of SPL than rodent beta-cells and rat islets [31]. This correlates with their delayed cytokine toxicity [50]. It will be important to see if suppression of SPL can accelerate cytokine toxicity in human beta-cells. In the context of glucolipotixicty, SPL seems to play an opposite role, at least under 24 h incubation with FFAs [31]. It accelerates FFA induced apoptosis; via depletion of lipid droplets and induction of oxidative stress [31]. It will be important to investigate whether these deleterious events are related to accumulation of proapoptotic ceramides and sphingomyelins. The role of SPP1 and SPP2 isoenzymes requires further investigations in vitro and in vivo, since SPP2 KO mice are characterized by defective beta-cell proliferation and ER stress [29]. SPP1 activity is needed for efficient recycling of sphingosine into the sphingolipid synthesis pathway [110]. The SPP2 expression is increased during the inflammatory response in many cell types [149] and is the predominant isoform in pancreatic beta-cells [30]. SPP1 has been shown to regulate autophagy induced by ER stress [150], and could be therefore engaged in the control of autophagy in beta-cells. Both SPP isoenzymes are also believed to regulate cell proliferation [29,146,151], and it would be important to see whether they are also involved in this process in beta-cells under diabetogenic conditions. Furthermore, the LPP protein family still needs to be characterized in beta-cells under T1DM- or T2DM-conditions. Currently no beta-cell specific mouse models exist and no data on the expression magnitude and pattern of enzymes of S1P metabolism in pancreas from diabetes models and in pancreatic sections from T1DM and T2DM donors are available.

The observed differences in the expression levels of S1P transporters and S1P receptors depending on the exposition time to proinflammatory cytokines indicate that shifting the S1P inside–out and activation of S1P receptors might be used as a regulatory mechanism in cytokine action and/or that differential changes may be involved signaling or toxic effects of cytokines.

The long-term effects of chronic exposure to FFAs, HG or cytokines on the expression pattern of S1P metabolizing enzymes in beta-cells and islets need characterization. Additionally, the expression of components of S1P metabolism in specimens of pancreata from T1DM and T2DM donors in comparison to healthy individuals need be investigated in order to verify the in vitro data relevance for human situation.

Moreover, the possible contribution of intra beta-cell generated S1P to immune cell attraction during T1DM development should be addressed. By controlling the intracellular S1P, SPL may regulate the amount of S1P available for export and thereby regulate the signaling through extracellular S1P receptors. S1P and S1PR signaling strongly regulate lymphocyte trafficking and survival [107,152,153,154]. Therefore, proinflammatory cytokine-mediated changes of the expression and enzymatic activities of S1P metabolizing enzymes as well of S1P receptors and transporters by regulating the amount of S1P transported inside–out by pancreatic beta-cells may participate in the attraction of immune cells and inflammation of islets in T1DM. Inflammation could be also promoted by intracellular S1P as described in other cell types [6,138,155], by mechanisms involving the activation of STAT3 and upregulation of specific microRNAs [113,156,157,158], as well as by generation of proinflammatory mediators [15,16,146]. Finally, S1P has been shown to epigenetically modulate gene expression in various cell types [130,131] and this aspect of S1P biology might be important for cytokine as well as FFA toxicity in beta-cells as well.

## 10. Conclusions and Perspectives

**Figure 2:**
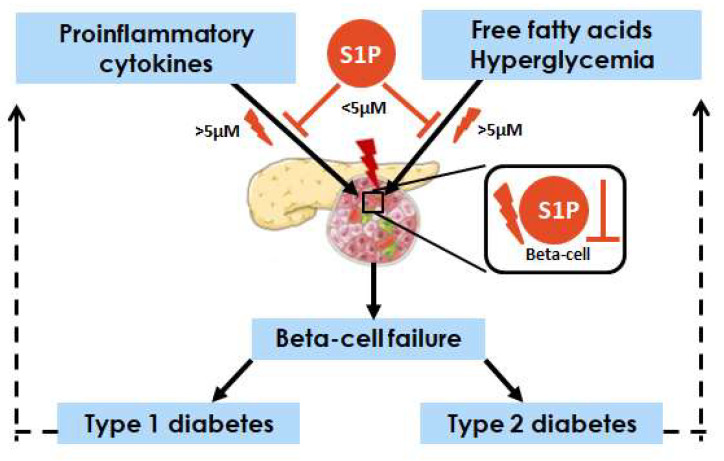
Involvement of sphingosine-1 phosphate (S1P) in beta-cell failure in T1DM and T2DM. Data gained from rodent and murine beta-cells suggest that extracellular S1P (at concentrations <5 µM) predominantly protects against cytokine and FFA-induced toxicity (survival and function), though at higher concentrations (>5 µM) it seems to negatively modulate cell viability and GSIS. Short-time exposure to proinflammatory cytokines (15 min–8 h) and PA (<24 h) increase S1P generation in beta-cells. The role of intracellular S1P remains unclear. Overexpression of SK1 leading to increased intracellular S1P generated in cytoplasm and in proximity of cell membrane has been associated with protective effects against PA toxicity. However, SK1 is weakly expressed in beta-cells (rodent, murine and human) and the predominant isoform is SK2. SK2-derived S1P has been shown to be involved in lipotoxicity, but its role in cytokine toxicity has not yet been addressed. Under short exposures (24 h), SPL overexpression seems to protect against cytokine-mediated toxicity, while it accelerates PA toxicity. The high expression of SPL in human beta-cells correlates with OA toxicity.

The regulation of S1P biosynthesis, recycling and degradation in pancreatic beta-cells remains largely under-investigated, particularly in human beta-cells. The effects of exogenous and intracellular S1P vary significantly depending on local concentrations of S1P and timing of exposure to diabetogenic conditions. In vivo these regulatory effects of S1P are expected to be prone to even larger changes due to interactions with other pro-, but also anti-inflammatory factors and dietary metabolites. At low concentrations, exogenously added S1P shows protective effects against cytokine toxicity and lipotoxicity (Figure 2), however at the higher concentrations S1P seems to negatively modulate beta-cell viability and GSIS. The effects of chronic exposure to S1P have not yet been investigated.

The effects of intracellular S1P seem to strongly depend on the localization of S1P biosynthesis, its duration and the activity of recycling and/or degrading mechanisms. Thus far, it has been demonstrated that downregulation of S1P concentration by SPL overexpression protects rodent beta-cells against acute cytokine toxicity (30), though whether SPL overexpression prevents cytokine toxicity also in the case of long-term exposure to cytokines has not been evaluated so far. This is in contrast to the toxic outcome of SPL overexpression on FFA-mediated toxicity in rodent beta-cells [31]. The deleterious effect of SPL overexpression in beta-cells exposed to lipotoxic conditions is in line with protective effects of SK1 overexpression [25,28]. It is important to state that these findings need to be verified under conditions of chronic exposure (>24 h), particularly in human beta-cells, and by addressing the role of other enzymes involved in S1P metabolism.

Thus, it will be important to perform detailed studies on molecular mechanisms involved in S1P action in beta-cells, particularly those focused on inflammation and epigenetic regulation. Exposure of existing SK1, SK2, SPP or SPL global KO animal models to STZ, generation of beta-cell specific S1P-metabolizing enzymes’ knock-out and knock-in mouse models, or development of beta-cell specific S1P-metabolizing enzymes’ knockouts in mouse models of diabetes, will advance our understanding of diabetes development mechanisms.

Of note, the expression of sphingosine kinases seems to be similar in rodent INS1E and human EndoC-βH1 beta-cells, however both cell lines differ in the expression magnitude of SPL [30,31]. These findings point to important differences in the S1P metabolism and—perhaps—action between rodent and human beta-cells and urge further characterization of the sphingolipid rheostat as well as S1P receptor and transporter systems in human beta-cells.

Finally, the characterization of the S1P metabolizing enzymes in human pancreata from nondiabetic and diabetic donors would enable understanding changes of S1P metabolism for diabetes development and considering modulators of S1P metabolism as potential therapeutic targets for clinical use in treatment of diabetes.

## Figures and Tables

**Table 1:** Effects of extracellular S1P in beta-cells and islets in the absence or presence of proinflammatory cytokines or FFAs. The summary is based mainly on data gained in rat and mouse beta-cells and islets. Details are discussed in Section 7.1 and Section 8.1. Ref: References to the studies.

Conditions	Effects	Ref.
Extracellular S1P alone	Nontoxic nM-5 µM < 24 h >5 µM inhibition of cell viability and GSIS >5 µM induction of Caspase-3 activation > 5µM inhibition of proliferation 5 µM weak induction of NFκB nM no induction of iNOS and NO Increase in cAMP No effect on ATP content Insulin secretion stimulation at 3 mM Glc Potentiation of GSIS (<5 µM) HDL-mediated inhibition of TUNEL staining after IL-1β or HG (mouse and human islets) S1P receptors specific (major role of S1P2 and S1P3) (MIN6, mouse islets; INS1, rat islets)	[25,26,30,105] [26,30] [26,30,105] [26,30] [30] [105] [30] [30] [30] [24,26,27,30] [141] [26,106,142]
Extracellular S1P and proinflammatory cytokines	Prevention of caspase-3 activation (INS1E) Decrease in TUNEL staining (INS1, rat islets) Inhibition of apoptosis (MIN6) Prevention of cytochrome c release (INS1) Prevention of viability loss (INS1E) Partial protection from proliferation inhibition (INS1E) No additive effect on NFκB (INS1E) No additive effect on iNOS and NO (INS1) S1P2 and S1P3-mediated effects (MIN6, mouse islets, INS1, rat islets) PKC-mediated effects (INS1)	[30,105] [105] [26] [105] [30] [30] [30] [105] [26,105] [142] [106,142]
Extracellular S1P and FFAs	Prevention of PA-mediated apoptosis (double staining with annexin V and propidium iodide, caspase-3 activation, TUNEL staining) (INS1, MIN6, mouse islets) Prevention of PA-mediated cell death (PI) (MIN6)	[26,28] [28]

**Table 2:** Effects of S1P-metabolizing enzymes and intracellular S1P on toxicity of proinflammatory cytokines and FFAs in beta-cells. Shown are effects of cytokines and PA on S1P metabolizing enzymes in rodent beta-cells (no data on other FFAs effects so far) and consequences of genetic manipulations of these enzymes considering susceptibility to cytokines and FFAs (PA or OA) in rodent and human beta-cells. The summary is based on observations made within a short-term exposure to cytokines and FFAs (up to 24 h); the effects of chronic changes of intracellular S1P on cytokine or PA-mediated toxicity have not yet been studied. The role of intracellular S1P in cytokine toxicity is described on the basis of so-far limited observations made in rat INS1E cells with genetically modified expression of SPL. Currently no studies on the effects of SK1 or SK2 genetic modifications on cytokine toxicity in beta-cells are available. Only effects of SPL, but not of SK1 or SK2, on FFA-mediated toxicity have been analyzed in human beta-cells. Of note, high SPL expression correlates with OA toxicity in human beta-cells. Ref.: References to studies.

Enzymes and S1P	Proinflammatory cytokines	Ref.	FFAs	Ref.
SK1	Expression upregulated (1-8 h) Expression unaffected (24 h) Activity unaffected (1-24 h) Function not studied	[106] [30] [105,106]	Expression upregulated by PA (24 h) Activity upregulated by PA Overexpression protective against PA Suppression toxic against PA	[25] [26,27] [25] [25,28]
SK2	Expression unaffected (1-8 h) Expression upregulated (24 h) Activity upregulated (15 min-8 h) Function not studied	[106] [30] [105,106]	Expression unaffected by PA (24 h) Expression upregulated by PA (24 h) Activity upregulated by PA? Overexpression deleterious against PA	[25] [132] [26,27] [132]
SPL	Expression mildly upregulated (6h) Expression downregulated (24 h) Overexpression protective (24 h) Suppression deleterious (24 h)	[30] [30] [30] [30]	Expression-no data Overexpression toxic against PA (24 h) Overexpression induces OA toxicity (24 h) Suppression protective in EndoC-βH1 cells (24 h)	[31] [31] [31]
S1P function	Based on SPL overexpression studies in INS1E cells S1P participates in early phase of cytokine toxicity (24 h):		Based on SK1 and SPL studies S1P protects against FFA toxicity; with exception of SK2-derived S1P that is deleterious (24 h):	
	Increase of S1P, but no release from cells Cell viability inhibition Caspase-3 activation Proliferation inhibition CHOP upregulation Calcium homeostasis disruption Mitochondrial chaperones down ATP content fall BAD dephosphorylation No effects on ROS generation GSIS inhibition	[106] [30] [30] [30] [30] [30] [30] [30] [30] [30] [30]	Increase of S1P and release from cells Cell viability Inhibition of apoptosis ER stress downregulation Inhibition of oxidative stress Prevention of ceramide generation Mitochondrial chaperones up Inhibition of cytochrome c release Lipid droplet formation GSIS potentiation	[25,26,105] [31] [25,28] [31] [31] [25] [31] [28] [31] [25,26,27,28,132]

## Data Availability

The data presented in this study are available on request from the corresponding author.
